# Study on Echinacoside-Copper Metal–Phenolic Networks Hydrogel System Loaded with Diallyl Trisulfide for Local Treatment of Cervical Cancer

**DOI:** 10.3390/pharmaceutics18091126

**Published:** 2026-09-08

**Authors:** Na Zhao, Xiaoqian Zhang, Jing Luo, Xiaoyue Zhang, Yonghong Zhao, Jiang Liu, Le Li, Chenglin Hong, Shiguo Sun

**Affiliations:** 1 Key Laboratory of Xinjiang Phytomedicine Resource and Utilization, Ministry of Education, Institute for Safflower Industry Research, College of Pharmacy, Shihezi University, Shihez 832002, China; zhaona@shzu.edu.cn (N.Z.); 20232015021@stu.shzu.edu.cn (X.Z.); 15699399805@163.com (J.L.); 15160973823@163.com (X.Z.); 13709930124@163.com (Y.Z.); liujiang@shzu.edu.cn (J.L.); teacher.lee@foxmail.com (L.L.); 2 College of Chemistry & Pharmacy, Northwest A&F University, Xianyang 712100, China

**Keywords:** cervical cancer, metal–phenolic networks, echinacoside, diallyl trisulfide, thermosensitive hydrogel

## Abstract

**Background/Objectives:** Cervical cancer cells evade chemotherapy by upregulating antioxidant defenses (e.g., glutathione (GSH)). A platform that simultaneously amplifies ROS and suppresses antioxidant defense is a low toxicity strategy. This study presents a local nanoplatform that combines chemodynamic therapy (CDT) with chemotherapy for the localized treatment of cervical cancer. **Methods:** Diallyl trisulfide (DATS) was loaded into the echinacoside (ECH)-copper metal–phenolic networks (MPNs) nanoparticles (ECD NPs) through a one-step coordination assembly method. The nanoparticles were incorporated into a poloxamer/HPMC thermosensitive hydrogel for vaginal delivery. The formulation was characterized for size, drug loading, sol–gel transition, and pH-responsive release. Antitumor activity was evaluated in SiHa and HeLa cells via Cu^2+^ uptake, GSH depletion, ROS accumulation, apoptosis markers, and viability. In vivo efficacy and biosafety were assessed in an orthotopic cervical cancer model. **Results:** ECD NPs showed uniform size (~135 nm), high DATS loading (~27.6%). The ECD NPs-loaded hydrogel exhibited a sol–gel transition at 36.7 °C. The ECD NPs-loaded hydrogel released 16.4% of DATS at pH 7.4, 32.4% at pH 4.5, 64.8% at pH 6.5, and 80.9% at pH 5.6 over 24 h. Release was minimal at vaginal pH, clearly triggered at tumor pH, and fastest at lysosomal pH, confirming pH-responsive behavior. The ECD NPs-loaded hydrogel enhanced Cu^2+^ uptake, depleted GSH, elevated ROS, and reduced cell viability to <50% at 80 μg/mL. In the orthotopic model, the ECD NPs hydrogel achieved a tumor inhibition rate of 87.81%, significantly outperforming free DATS (65.3%) and blank MPN (55.62%) hydrogels, with no evident systemic toxicity. **Conclusions:** The ECD NPs hydrogel triggers a Cu^2+^-driven ROS/GSH cascade that combines copper-mediated oxidative stress with DATS-induced apoptosis for enhanced antitumor activity. Its vaginal localization, pH-responsive release, and biosafety profile support further evaluation for cervical cancer therapy.

## 1. Introduction

Cervical cancer is the fourth most common malignancy among women worldwide and a leading cause of cancer-related deaths, with approximately 660,000 new cases and 350,000 deaths estimated in 2022 [1,2]. Although the widespread adoption of Human Papillomavirus (HPV) vaccines and advances in screening techniques have improved the prevention and control of cervical cancer, the disease burden remains disproportionately high in low- and middle-income regions [3,4]. A further compounding trend is the steady rise in cervical cancer incidence among younger women of reproductive age [5,6]. For this patient population, conservative surgical options such as radical trachelectomy or neoadjuvant platinum-based chemotherapy are the primary options. However, each approach has considerable limitations. Radical surgery risks reproductive organ damage and postoperative complications. Systemic chemotherapy often causes severe, dose-limiting toxicities, such as myelosuppression, neurotoxicity, and gonadotoxicity, which can irreversibly impair ovarian function and fertility [7,8]. These drawbacks underscore the demand for localized therapeutic strategies that provide potent antitumor efficacy while minimizing reproductive toxicity.

The accessible anatomical position of the cervix permits direct vaginal drug delivery, bypassing the systemic circulation and achieving high local drug concentrations [9,10,11]. Hydrogels have gained attention for localized cervical cancer therapy due to their biocompatibility, porous structure, and sustained release capacity [12,13,14]. Recent studies have demonstrated that rationally designed hydrogels can achieve effective treatment of cervical cancer with minimal off-target toxicity [14]. These properties prolong tumor retention and reduce systemic exposure. Among them, polyoxyethylene-based thermosensitive hydrogels, such as poloxamer-based formulations, offer distinct advantages. These systems are free-flowing sols at room temperature, which facilitates convenient and minimally invasive administration. At body temperature, they rapidly transform into a semi-solid gel that adapts to irregular mucosal surfaces [15,16,17]. Despite these advantages, conventional thermosensitive hydrogels function largely as passive depots. They do not respond to tumor microenvironment (TME) cues such as mild acidity, which can result in premature drug leakage and suboptimal therapeutic outcomes.

The choice of therapeutic agent is equally critical. Diallyl trisulfide (DATS) is a bioactive organosulfur compound derived from garlic [18,19,20,21,22]. DATS exhibits antitumor activity against cervical cancer, which is potentially associated with the modulation of key signaling pathways involved in cell proliferation and apoptosis [23,24,25]. However, its clinical translation is severely hindered by high volatility, chemical instability, poor aqueous solubility, and an irritating odor [26,27]. A critical limitation of DATS-based therapy is that the resulting oxidative stress is frequently offset by endogenous antioxidant systems. Cancer cells maintain high glutathione (GSH) levels, which efficiently neutralize reactive oxygen species (ROS) and thereby suppress apoptosis [28]. To overcome these obstacles, stimuli-responsive nanoplatforms that actively disrupt redox homeostasis are being explored [29,30,31,32]. Metal–phenolic networks (MPNs) are formed by the self-assembly of metal ions and polyphenol ligands [33,34]. They represent a versatile platform for drug delivery and chemodynamic therapy (CDT) [35,36,37]. In the acidic TME, MPNs dissociate, releasing metal ions like Cu^2+^ that catalyze Fenton-like reactions to generate highly cytotoxic hydroxyl radicals (·OH) from endogenous H_2_O_2_ while simultaneously depleting GSH [38,39,40,41]. Copper-based MPNs have been reported to disrupt redox homeostasis through ROS generation and GSH consumption [42,43]. However, most reported MPNs rely on conventional polyphenols like tannic acid or gallic acid with Fe^3+^, which primarily serve as structural scaffolds and lack intrinsic therapeutic activity [44,45,46]. This presents a clear opportunity to design a more functional MPN system leveraging a bioactive polyphenol.

Herein, we report a multifunctional localized nanoplatform designed to address these challenges. The system integrates a natural polyphenol with intrinsic pharmacological activity, the antitumor agent DATS, and Cu^2+^-based CDT within a single tumor-responsive delivery vehicle. Echinacoside (ECH) is a phenylethanoid glycoside derived from *Cistanche deserticola* [47]. It exhibits intrinsic antitumor properties [48,49,50,51,52]. We hypothesize that ECH can act as a dual-functional ligand for the construction of Cu^2+^-based MPNs [53,54,55,56]. This ECH-Cu^2+^ MPN would not only act as a pH-responsive nanocarrier but also contribute to the overall therapeutic effect. DATS was encapsulated within the ECH-Cu^2+^ network to form ECH-Cu^2+^-DATS nanoparticles (ECD NPs). Upon vaginal delivery and tumor uptake, the nanoplatform was designed to combine Cu^2+^-mediated CDT, ECH-modulated apoptosis, and DATS-induced mitochondrial apoptosis. In this design, Cu^2+^-mediated CDT generates ·OH and depletes GSH, thereby sensitizing cancer cells to oxidative damage. ECH concurrently modulates apoptotic pathways, while DATS triggers mitochondrial apoptosis. Collectively, these complementary actions are expected to enhance antitumor efficacy.

**Scheme 1:**
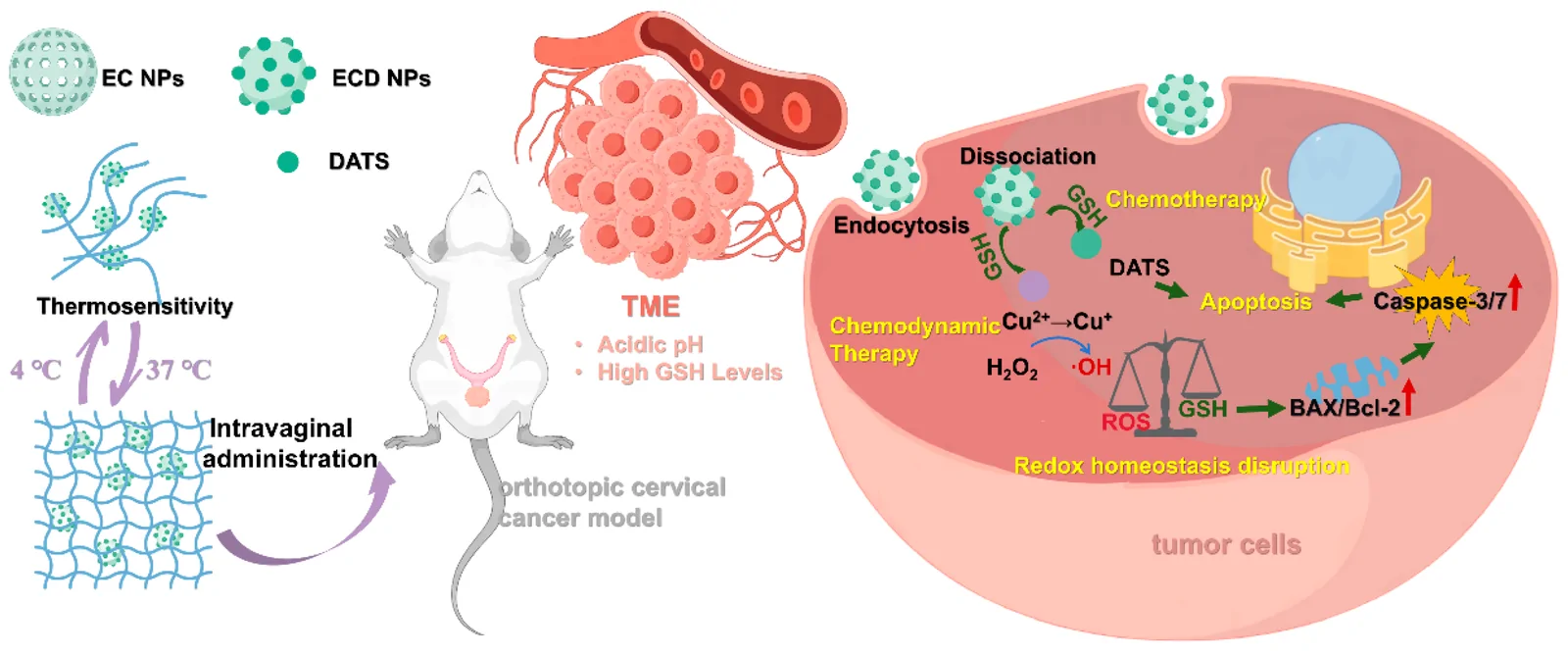
A schematic illustration of the preparation of the ECD NPs-loaded thermosensitive hydrogel and its local administration for cervical cancer therapy.

To enable localized and sustained delivery, these ECD NPs are incorporated into a poloxamer/hydroxypropyl methylcellulose (HPMC) thermosensitive hydrogel tailored for vaginal administration. The sol–gel transition temperature was tuned to 36.7 °C, close to physiological conditions. This ensures rapid solidification upon vaginal application. The ECH-Cu^2+^ coordination enables pH-responsive drug release. Drug leakage is limited at normal vaginal pH (~4.5), while rapid release is triggered under acidic conditions (pH 6.5 for tumor extracellular environment and pH 5.6 for lysosomal compartment) [57,58,59]. As shown in Scheme 1, this design was motivated by three persistent challenges in cervical cancer therapy. First, conventional local delivery systems inadequately balance tumor-specific accumulation with systemic toxicity reduction [10,14]. Second, single-agent regimens frequently yield suboptimal clinical responses due to intrinsic or acquired resistance [3,18]. Third, combination strategies capable of concurrently disrupting pro-survival and anti-apoptotic pathways remain underdeveloped [28]. In this study, the physicochemical properties of the ECD NPs and the hydrogel were characterized, the enhanced antitumor mechanism was evaluated in SiHa and HeLa cervical cancer cells, and the therapeutic efficacy and biosafety of the system were assessed in an orthotopic mouse model. These findings suggest that the ECD NPs-loaded thermosensitive hydrogel represents a promising candidate for local treatment of cervical cancer.

## 2. Materials and Methods

### 2.1. Materials

Echinacoside (ECH, CAS No. 82854-37-3, HPLC purity ≥ 98%), diallyl trisulfide (DATS, CAS No. 2050-87-5, GC purity ≥ 98%), and copper chloride dihydrate (CuCl_2_·2H_2_O, purity ≥ 99%) were purchased from Shanghai Macklin Biochemical Technology Co., Ltd. (Shanghai, China). Poloxamer 407 (P407, pharmaceutical grade), poloxamer 188 (P188, pharmaceutical grade), and hydroxypropyl methylcellulose (HPMC, viscosity 4000 mPa·s) were obtained from Xi’ an Tianzheng Pharmaceutical Excipients Co., Ltd. (Xi’an, China). HPLC-grade methanol and formic acid were purchased from Merck KGaA (Darmstadt, Germany). DMEM high-glucose medium, fetal bovine serum (FBS), and penicillin–streptomycin solution were obtained from Thermo Fisher Scientific (Waltham, MA, USA). Trypsin–EDTA digestion solution and thiazolyl blue tetrazolium bromide (MTT) were purchased from Beijing Solarbio Science & Technology Co., Ltd. (Beijing, China). The Annexin V-FITC/PI apoptosis detection kit, reduced GSH assay kit, reactive oxygen species (ROS) assay kit, and cellular copper assay kit were all obtained from Beijing Solarbio Science & Technology Co., Ltd. (Beijing, China). The hematoxylin and eosin (H&E) staining kit and the terminal deoxynucleotidyl transferase dUTP nick end labeling (TUNEL) apoptosis detection kit were purchased from Beijing Solarbio Science & Technology Co., Ltd. (Beijing, China).

### 2.2. Cell Lines and Animals

Human cervical cancer cell lines SiHa and HeLa were obtained from the Cell Resource Center, Institute of Basic Medical Sciences, Chinese Academy of Medical Sciences (Beijing, China). Mouse U14 cervical cancer cells were purchased from Suzhou Haixing Biotechnology Co., Ltd. (Suzhou, China). SiHa and HeLa cells were cultured in DMEM/high glucose medium supplemented with 10% fetal bovine serum (FBS) and 1% penicillin–streptomycin. U14 cells were maintained in DMEM-H medium containing 10% FBS and 1% penicillin–streptomycin. All cells were cultured in a humidified atmosphere at 37 °C with 5% CO_2_. Cells in the logarithmic growth phase were used for all experiments. Regular mycoplasma testing was performed and yielded negative results.

Female Kunming (KM) mice (4–6 weeks old, weighing 18–22 g) were obtained from Beijing Sibef Biotechnology Co., Ltd. (Beijing, China). All the animals were housed in a specific pathogen-free (SPF) facility under controlled environmental conditions: temperature (22 ± 2) °C, relative humidity (55 ± 5) %, and a 12 h light/dark cycle. The animals had ad libitum access to standard chow and water. All animal procedures were approved by the Animal Ethics Committee of Shihezi University (Approval No. A2026-805, approved March 2026) and complied with the NIH Guide for the Care and Use of Laboratory Animals.

### 2.3. Preparation and Optimization of EC NPs and ECD NPs

ECH-Cu^2+^ MNPs nanoparticles (EC NPs) were prepared via coordination self-assembly. Briefly, ECH was dissolved in a mixture of deionized water and dimethyl sulfoxide (DMSO) (1:1, *v*/*v*) to obtain a 16 mM ECH solution. CuCl_2_·2H_2_O was dissolved in deionized water to prepare a 16 mM Cu^2+^ stock solution. Subsequently, 3 mL of the ECH solution was placed in a beaker under magnetic stirring at 500 r/min, and 3 mL of the CuCl_2_ solution was slowly added dropwise, followed by continuous stirring for 2 h. After the reaction, the mixture was centrifuged at 6000 r/min for 10 min to remove precipitates, and the supernatant was further centrifuged at 15,000 r/min for 10 min to collect the precipitate. The obtained precipitate was redispersed in deionized water, ultrasonicated for 3 min, and washed by repeated centrifugation three times. The final product was lyophilized to obtain EC NPs.

ECD NPs were prepared based on the above procedure with the addition of 6 mL of 32 mM DATS solution in DMSO. The remaining steps were identical to those used for EC NP preparation, and the final product was lyophilized for subsequent use.

Single-factor optimization experiments were conducted to determine the optimal preparation parameters. Three mixing methods were compared: one-step mechanical stirring, ultrasonic bath treatment, and probe sonication (using a cell sonicator). In addition, the molar ratio of ECH to Cu^2+^ was optimized over a range of 1:1, 2:1, 3:1, 4:1, 5:1, and 6:1. The optimal conditions were determined based on nanoparticle size and polydispersity index (PDI) measured by dynamic light scattering.

### 2.4. Characterization of Physicochemical Properties of Nanoparticles

Particle size and zeta potential measurement: EC NPs and ECD NPs were diluted to appropriate concentrations with deionized water and equilibrated at 25 °C for 2 min prior to measurement. The particle size, PDI, and zeta potential were determined using a Malvern laser particle size analyzer. Each sample was measured in triplicate and data are presented as mean ± SD.

Morphological characterization: EC NPs and ECD NPs were diluted and dropped onto copper grids, dried under an infrared lamp, and observed using transmission electron microscopy (TEM) to examine their morphology.

Chemical structure characterization: The infrared spectra of EC NPs, ECD NPs, ECH, and DATS were measured using a Fourier transform infrared (FTIR) spectrometer in the scanning range of 4000–400 cm^−1^ to analyze their chemical interactions.

Determination of drug loading capacity and encapsulation efficiency: An HPLC method was established to determine the content of DATS. The chromatographic conditions were as follows: an octadecylsilyl silica gel column (250 mm × 4.6 mm, 5 μm) as the stationary phase, a mobile phase consisting of methanol-0.1% formic acid solution (75:25, *v*/*v*), a detection wavelength of 210 nm, a flow rate of 1.0 mL/min, and a column temperature of 30 °C. An appropriate amount of ECD NPs was accurately weighed, dissolved and diluted to volume with anhydrous ethanol. After centrifugation, the supernatant was injected for analysis. Method validation confirmed good linearity (R^2^ = 0.9998, 10.4–166.4 μg/mL), precision (RSD < 0.1%), repeatability (RSD < 1.0%), stability (RSD < 0.6% over 24 h), and recovery (97.3–103.2%, RSD < 2.0%). Representative HPLC chromatograms are shown in Figure S1, and the detailed validation parameters are summarized in Table S1. The drug loading capacity was calculated using the following formula:

Drug loading capacity (%) = (mass of DATS in nanoparticles/total mass of nanoparticles) × 100%

All measurements were performed in triplicate (*n* = 3).

### 2.5. Preparation and Characterization of ECD NPs-Loaded Hydrogel

The thermosensitive hydrogel matrix was prepared following our previously established formulation. The concentrations of P407, P188, and HPMC were systematically optimized to achieve a sol–gel transition temperature close to physiological conditions [15]. Briefly, the matrix composed of P407 (20%, *w*/*v*), P188 (18%, *w*/*v*), and HPMC (0.5%, *w*/*v*) was dissolved in deionized water and heated in a 60 °C water bath until completely dissolved. After cooling to room temperature, an appropriate amount of ECD NPs lyophilized powder was added and uniformly dispersed by ultrasonication to obtain the ECD NPs-loaded hydrogel.

Scanning electron microscopy (SEM) measurement: To observe the internal microstructure of hydrogels, samples were rapidly frozen in liquid nitrogen. Then, the samples were completely dried in a freeze dryer. The dried samples were sputter coated with gold. Finally, the morphology was examined and documented by SEM at 3.0 kV.

Rheological measurement: The storage modulus (G′) and loss modulus (G″) of the hydrogel were measured using a rotational rheometer to investigate its rheological properties.

In vitro degradation study: The hydrogel was placed in PBS buffer (pH 5.6) and incubated at 37 °C with constant shaking. Samples were taken at predetermined time points, the mass change of the hydrogel was measured, and the degradation rate was calculated.

Hemocompatibility assay: Fresh mouse blood was collected to prepare a red blood cell suspension, which was incubated with different concentrations of ECD NPs. After centrifugation, the absorbance of the supernatant was measured to calculate the hemolysis rate, evaluating the hemocompatibility of the formulation.

### 2.6. In Vitro Drug Release Study

The in vitro release of DATS from different formulations was investigated using the dialysis bag method. Dialysis bags (MWCO 3.5 kDa) were pretreated by boiling in deionized water for 10 min to remove preservatives prior to use. Various formulations including ECD NPs-loaded hydrogel at pH 4.5, 5.6, 6.5, and 7.4; ECD NPs solution without hydrogel at pH 5.6; and free DATS-loaded hydrogel at pH 5.6 were evaluated. Each sample containing an equivalent amount of DATS was transferred into dialysis bags and immersed in PBS buffer at the corresponding pH. All samples were incubated at 37 °C with constant shaking at 100 r/min. At predetermined time intervals, aliquots of the release medium were withdrawn and replaced with an equal volume of fresh buffer. The DATS content in the release medium was determined by HPLC, and the cumulative release rate was calculated.

### 2.7. In Vitro Antitumor Experiments

MTT assay: SiHa and HeLa cells in the logarithmic growth phase were seeded into 96-well plates. After 24 h of culture, cells were treated with different concentrations of free DATS-loaded hydrogel, ECD NPs-loaded hydrogel, and incubated for an additional 24 h. The hydrogel formulations were stored at 4 °C prior to use. Immediately before addition to cells, each formulation was filtered through a 0.22 μm sterile syringe filter in a laminar flow cabinet, and then added to the cells in its sol state at room temperature. MTT reagent was then added to each well. Following 4 h of incubation, the formazan crystals were dissolved in DMSO, and the absorbance was measured at 490 nm using a microplate reader. Cell viability was calculated.

Cell morphology assay: SiHa and HeLa cells were seeded in 6-well plates at 1 × 10^5^ cells/well. After 24 h, the cells were treated with the blank control, EC-loaded hydrogel, DATS-loaded hydrogel, or ECD NPs-loaded hydrogel at 80 μg/mL for 24 h. Cell morphology was observed under an inverted phase-contrast microscope.

Apoptosis assay: SiHa and HeLa cells were seeded in 6-well plates, cultured for 24 h, and then treated with the blank control, EC NPs-loaded hydrogel, DATS-loaded hydrogel, or ECD NPs-loaded hydrogel for 24 h. After harvesting and washing, the cells were stained with Annexin V FITC and propidium iodide (PI), and apoptosis was assessed by flow cytometry using the Annexin V FITC/PI Apoptosis Detection Kit.

Mechanism-related assays: The Cu^2+^ uptake, GSH content, and ROS levels in SiHa and HeLa cells after blank control, EC NPs-loaded hydrogel and ECD NPs-loaded hydrogel treatment were determined using the Cu^2+^ Assay Kit, GSH Assay Kit, and ROS Assay Kit, respectively.

BAX and Bcl-2 levels were measured by ELISA, normalized to total protein, and expressed as the BAX/Bcl-2 ratio. Caspase-3/7 activity was measured at 24 h using a colorimetric assay with Ac-DEVD-pNA substrate. The 24 h time point was chosen to align with the MTT and apoptosis assays for consistent comparison across experiments, and is consistent with previous reports measuring caspase-3/7 activity in cervical cancer cells [60,61]. All assays were performed in triplicate, and results were presented as fold change relative to controls after protein normalization.

### 2.8. In Vivo Antitumor Experiment

Establishment of orthotopic cervical cancer mouse model: Mice were acclimatized for 1 week, then anesthetized with pentobarbital sodium (50 mg/kg, i.p.). U14 cells (1 × 10^6^ in 100 μL PBS) were injected intravaginally into the cervical submucosa. After 7 days, tumor establishment was verified by colposcopy, and mice bearing visible tumors (~100 mm^3^) were enrolled.

Animal grouping and administration: The successfully modeled mice were randomly divided into four groups (*n* = 5 per group): negative control group (normal saline), free DATS hydrogel group, EC NPs-loaded hydrogel group, and ECD NPs-loaded hydrogel group. The drugs were administered via the vaginal route once every three days. The dosage, calculated based on DATS content, was 20 mg/kg. The EC NPs-loaded hydrogel group was administered at the same nanoparticle mass as the ECD NPs group. The treatment lasted for 15 consecutive days.

In vivo efficacy evaluation: Body weight was measured every 3 days during treatment, and mortality was recorded daily. At the end of treatment, all mice were euthanized by cervical dislocation. The entire reproductive tract, including the cervix, uterus, and vagina, was then carefully dissected and excised. In situ tumor volume could not be assessed by external calipers owing to the deep anatomical location of the cervix. Accordingly, antitumor efficacy was evaluated by tumor weight measured ex vivo after careful dissection. The tumor inhibition rate was calculated using the following formula: Tumor inhibition rate (%) = (tumor mass of control group—tumor mass of treatment group)/tumor mass of control group × 100%. Tumor tissues were fixed, embedded in paraffin, sectioned, and subjected to hematoxylin and eosin (H&E) staining and TUNEL apoptosis staining. Tumor tissue morphology and apoptosis were observed under an optical microscope.

In vivo toxicity evaluation: After euthanasia, major organs including the heart, liver, spleen, lungs, and kidneys were collected, fixed, embedded in paraffin, sectioned, and stained with H&E. The pathological morphology of the organs was observed under an optical microscope to evaluate the in vivo toxicity of the ECD NPs hydrogel.

### 2.9. Statistical Analysis

All experimental data were expressed as mean ± standard deviation (SD). Statistical analysis was performed using SPSS 26.0 software. Differences among multiple groups were analyzed by one-way analysis of variance (ANOVA) followed by Tukey’s post hoc test for pairwise comparisons. Statistical significance was set at * *p* < 0.05, ** *p* < 0.01, *** *p* < 0.001.

## 3. Results

### 3.1. Preparation and Physicochemical Characterization of ECD NPs

**Figure 1:**
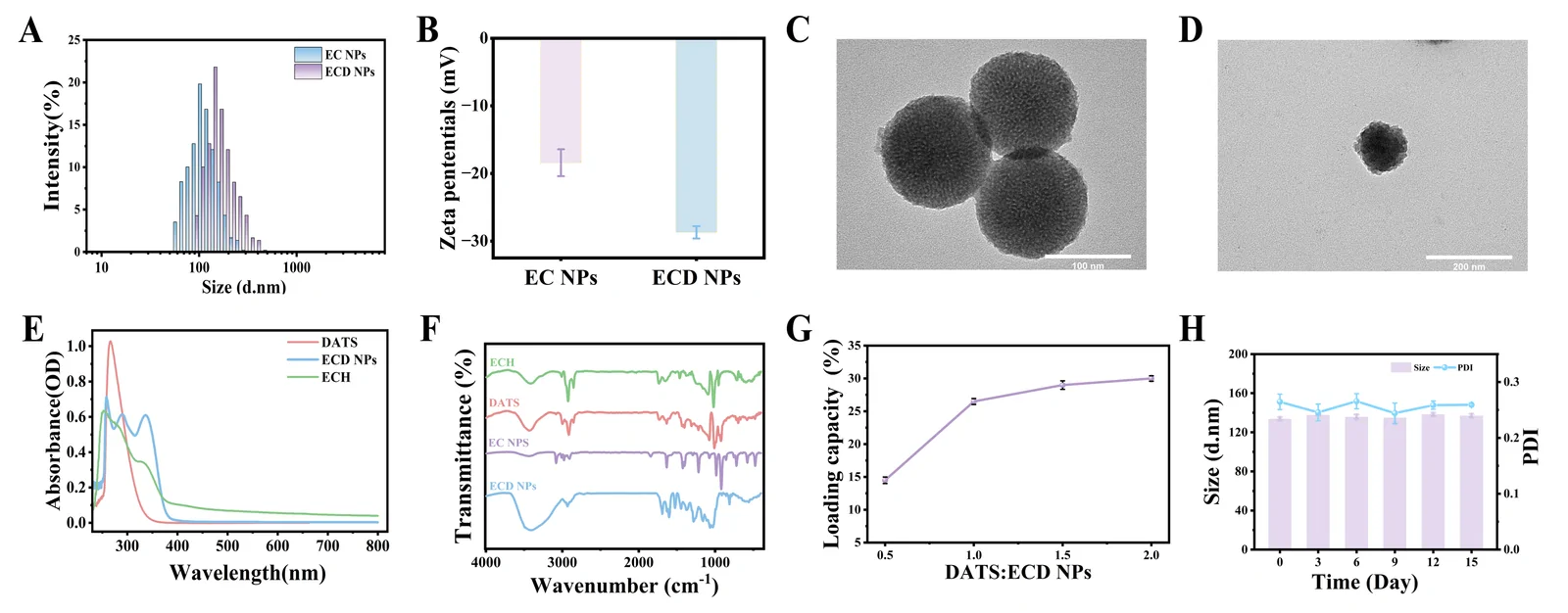
Characterization of EC NPs and ECD NPs. (**A**) Hydrodynamic diameter distributions of EC NPs and ECD NPs measured by dynamic light scattering. (**B**) Zeta potentials of EC NPs and ECD NPs. (**C**) TEM image of EC NPs showing spherical morphology and good monodispersity. (**D**) TEM image of ECD NPs after DATS loading. (**E**) UV-Vis absorption spectra of ECH, DATS, and ECD NPs. (**F**) FTIR spectra of ECH, DATS, EC NPs, and ECD NPs. (**G**) Drug loading capacity of ECD NPs at different DATS: Cu^2+^: ECH molar ratios. (**H**) Stability of ECD NPs over 15 days in deionized water, showing particle size and PDI. Data are presented as mean ± SD (*n* = 3).

The synthesis of EC NPs was optimized via a one-step stirring method. A molar ratio of ECH to Cu^2+^ of 2:1 was identified as optimal, yielding nanoparticles with minimal particle size and PDI (Figure S2). Under these conditions, the EC NPs had a hydrodynamic diameter of 118 ± 2 nm (PDI: 0.188 ± 0.02) and a zeta potential of −18 ± 1 mV (Figure 1A,B). The ECD NPs exhibited a diameter of 135 ± 5 nm (Figure 1A) and a zeta potential of −29 ± 0.85 mV (Figure 1B). The shift in surface charge following DATS encapsulation confirmed drug incorporation into the MPN matrix. TEM revealed that both EC NPs and ECD NPs possessed a regular spherical morphology with good monodispersity (Figure 1C,D). The porous-like morphology as observed by TEM in EC NPs became less distinct after drug loading, further corroborating the successful incorporation of DATS.

UV-Vis and FTIR spectroscopy both confirmed successful DATS loading. ECH alone showed two absorption peaks at 280 nm and 330 nm. ECD NPs exhibited a prominent enhanced peak at 210 nm (Figure 1E). This peak corresponds to the allyl sulfide bond of DATS. In FTIR analysis, the O-H stretching band of ECH at ~3400 cm^−1^ shifted to lower wavenumbers. Its intensity also flattened in EC NPs. This change indicated chelation between phenolic hydroxyl groups and Cu^2+^. ECD NPs further displayed two shoulder peaks at ~915 cm^−1^ and ~980 cm^−1^ (Figure 1F). These peaks match the terminal =CH_2_ groups of DATS. They were absent in pure ECH and blank EC NPs. Together, these data provide spectroscopic evidence for DATS encapsulation and MPN coordination. Quantification by HPLC revealed a high drug loading capacity of 27.6% at a DATS: Cu^2+^: ECH molar ratio of 2:2:1 (Figure 1G). Stability assessments demonstrated that ECD NPs maintained a consistent particle size (~135 nm) and PDI (<0.3) in deionized water over 15 days, indicating excellent colloidal stability (Figure 1H).

### 3.2. Gelation Behavior, pH-Responsive Degradation and Drug Release of ECD NPs-Loaded Hydrogel

**Figure 2:**
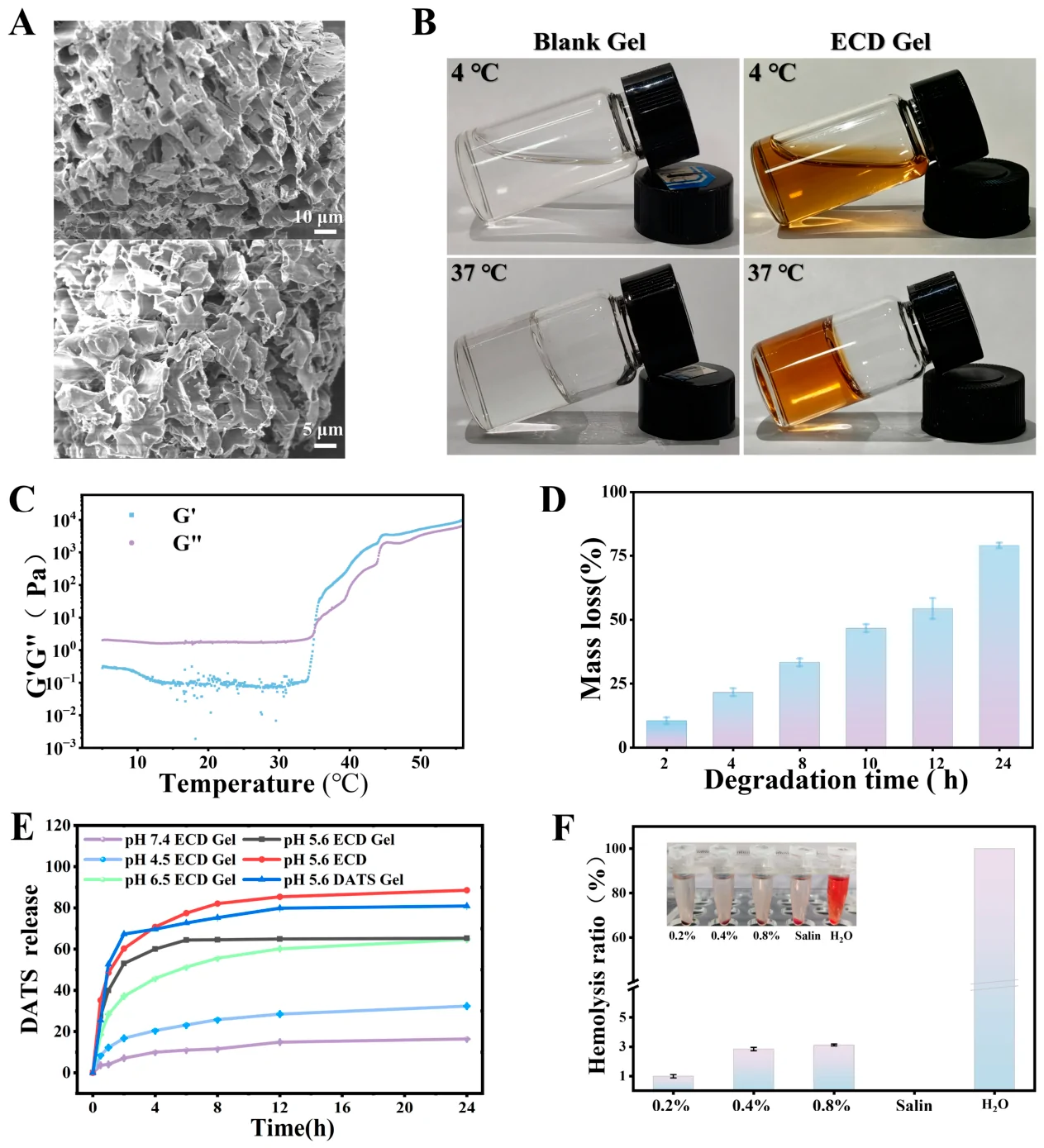
The physicochemical properties of the ECD NPs-loaded thermosensitive hydrogel. (**A**) A SEM image showing the three-dimensional porous network structure of the hydrogel. (**B**) Photographs of the hydrogel at 4 °C (sol state) and 37 °C (gel state). (**C**) Temperature-dependent changes in storage modulus (G′) and loss modulus (G″) of the hydrogel. (**D**) The in vitro degradation profile of the hydrogel at pH 5.6 over 24 h. (**E**) Cumulative release profiles of DATS from ECD NPs-loaded hydrogel at pH 7.4, 4.5, 6.5, and 5.6, along with ECD NPs solution (without hydrogel) and free DATS-loaded hydrogel at pH 5.6. (**F**) Hemolysis rates of the ECD NPs-loaded hydrogel at different concentrations (0.2–0.8%, *w*/*v*). The data represent mean ± SD from three independent measurements.

SEM revealed that the hydrogel matrix possessed a three-dimensional porous network structure with interconnected pores (Figure 2A). This porous structure provides a spatial basis for nanoparticle loading and drug release. The hydrogel was free-flowing at 4 °C and formed a semi-solid gel at 37 °C (Figure 2B). The hydrogel achieved a sol–gel transition at 36.7 °C with 3.5 mg/mL ECD NPs (Table S1), approximating the physiological temperature of the mouse vagina. The crossover point of G′ and G″ occurred at approximately 36 °C, beyond which G′ surpassed G″, confirming the formation of a viscoelastic gel network (Figure 2C).

In vitro degradation studies showed that the hydrogel degraded rapidly in a simulated tumor microenvironment (pH 5.6), reaching approximately 80% degradation within 24 h (Figure 2D). The release profiles of DATS from different formulations were evaluated at pH conditions covering the vaginal administration pathway. The ECD NPs-loaded hydrogel released 16.4% at pH 7.4, 32.4% at pH 4.5, 64.8% at pH 6.5, and 80.9% at pH 5.6 over 24 h. Minimal leakage occurred at normal vaginal pH 4.5, while tumor extracellular pH 6.5 triggered marked release, and lysosomal pH 5.6 gave the fastest release, favoring intracellular drug liberation. At pH 5.6, ECD NPs without hydrogel released 88.6% versus 80.9% for ECD NPs-loaded hydrogel, indicating a modest retarding effect of the gel matrix, while free DATS-loaded hydrogel released only 65.3%, confirming nanoparticle encapsulation improved release efficiency. These results demonstrate pH-responsive release suited for vaginal application. The hemolysis rate of the hydrogel remained below 5% across all tested concentrations (0.2–0.8%), confirming its good hemocompatibility for vaginal application (Figure 2F).

### 3.3. In Vitro Cytotoxicity and Apoptosis-Inducing Activity of ECD NPs-Loaded Hydrogel Against Cervical Cancer Cells

**Figure 3:**
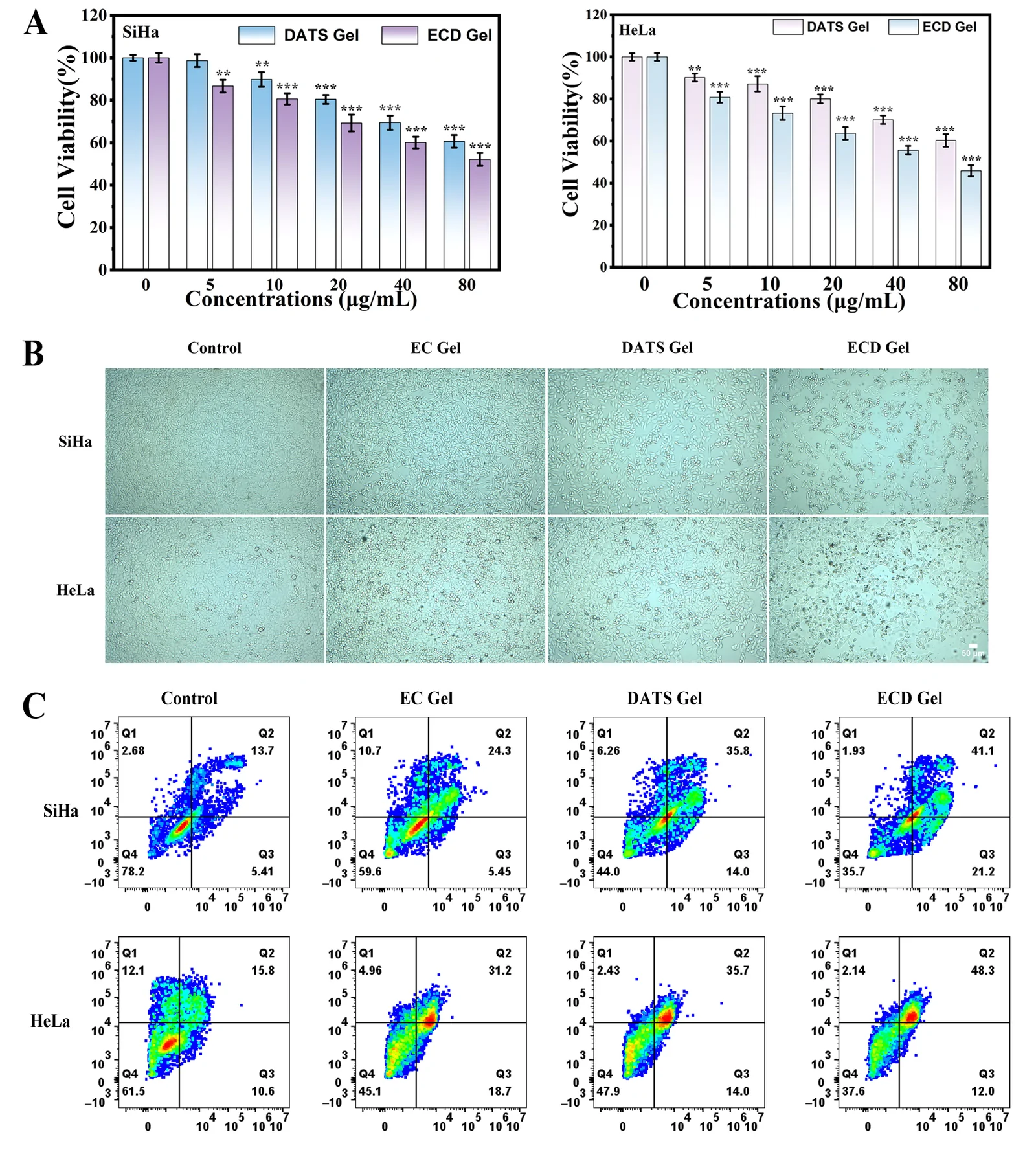
In vitro antitumor activity of ECD NPs-loaded hydrogel in cervical cancer cells. (**A**) Cell viability of SiHa and HeLa cells after 24 h treatment with different concentrations of ECD NPs-loaded hydrogel and free DATS hydrogel. (**B**) Morphological changes of SiHa and HeLa cells after 24 h treatment (scale bar = 50 μm). Cells were treated with control, EC NPs-loaded hydrogel, DATS-loaded hydrogel, or ECD NPs-loaded hydrogel. (**C**) Flow cytometric analysis of apoptosis in SiHa and HeLa cells after 24 h treatment with control, EC NPs-loaded hydrogel, free DATS-loaded hydrogel, and ECD NPs-loaded hydrogel. Data are presented as mean ± SD (*n* = 3). ** *p* < 0.01, *** *p* < 0.001 vs. untreated by Tukey’s HSD test after one-way ANOVA.

The antiproliferative activity of ECD NPs-loaded hydrogel was evaluated in SiHa and HeLa cells using an MTT assay. As shown in Figure 3A, ECD NPs-loaded hydrogel suppressed cell growth in a concentration-dependent manner. In SiHa cells, the cell viability ranged from 86.71% at 5 μg/mL to 52.13% at 80 μg/mL. In HeLa cells, the corresponding values decreased from 80.83% to 45.93% over the same concentration range. Free DATS-loaded hydrogel also exhibited concentration-dependent cytotoxicity, though less potent than ECD NPs hydrogel. At 80 μg/mL, the cell viability of free DATS-loaded hydrogel was 60.64% in SiHa cells and 60.34% in HeLa cells, both lower than those of ECD NPs hydrogel at the equivalent concentration. To visually support the MTT results, cell morphology was examined under an inverted phase-contrast microscope after 24 h treatment at 80 μg/mL. As shown in Figure 3B, control cells showed normal adherent growth with typical epithelial morphology. EC NPs-loaded hydrogel and DATS-loaded hydrogel groups exhibited modest cell rounding and detachment. ECD NPs-loaded hydrogel caused the most pronounced changes, including extensive cell shrinkage, rounding, detachment, and cellular debris. This order was consistent with the MTT results.

Apoptosis was assessed by Annexin V-FITC/PI double staining. Flow cytometric analysis revealed that the ECD NPs-loaded hydrogel induced total apoptosis rates of 64.3% in SiHa cells and 62.4% in HeLa cells after 24 h (Figure 3C). By comparison, the corresponding values for free DATS-loaded hydrogel were 56.0% and 52.1%, and for EC NPs-loaded hydrogel were 40.4% and 54.9%. The enhanced apoptosis in the ECD NPs-loaded hydrogel group, together with the elevated cytotoxicity observed in MTT assay, suggests a complementary combination effect between Cu^2+^-mediated oxidative stress and DATS-induced apoptosis in the nanoparticle platform.

### 3.4. Antitumor Mechanism: Cu^2+^ Uptake, GSH Depletion, ROS Elevation and Mitochondrial Apoptosis

**Figure 4:**
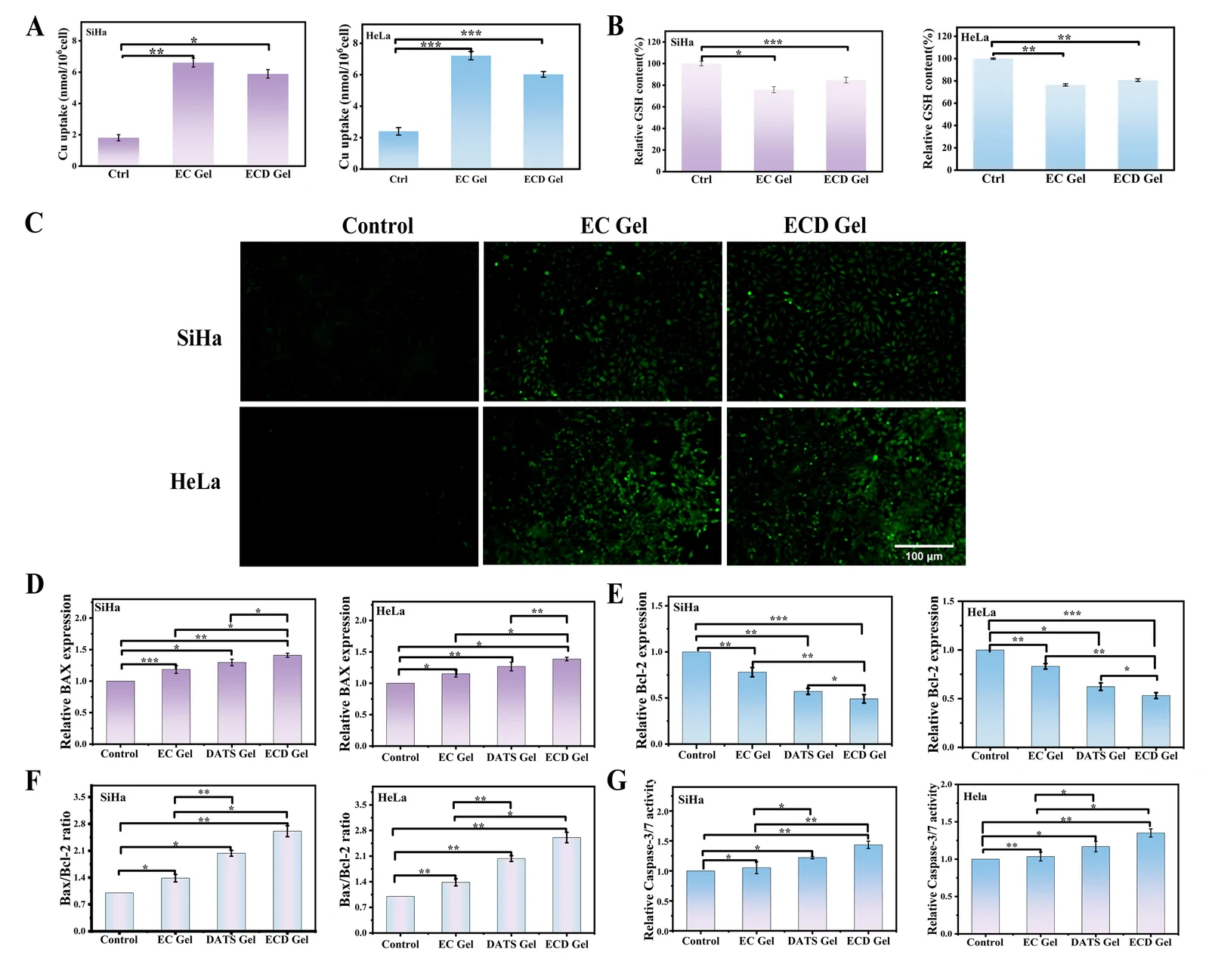
Synergistic antitumor mechanism of ECD NPs in cervical cancer cells. (**A**) Intracellular copper ion content in SiHa and HeLa cells after treatment with EC NPs and ECD NPs. (**B**) Relative GSH levels in SiHa and HeLa cells after different treatments. (**C**) Representative fluorescence images of ROS generation in SiHa cells (scale bar = 100 μm). (**D**) Relative BAX expression in SiHa cells and HeLa cells. (**E**) Relative Bcl-2 expression in SiHa cells and HeLa cells. (**F**) BAX/Bcl-2 ratio in SiHa cells and HeLa cells. (**G**) Relative caspase-3/7 activity in SiHa cells and HeLa cells. Cells were treated with control, EC NPs hydrogel, free DATS hydrogel, or ECD NPs hydrogel for 24 h. Data are presented as mean ± SD (*n* = 3). * *p* < 0.05, ** *p* < 0.01, *** *p* < 0.001 vs. untreated by Tukey’s HSD test after one-way ANOVA.

To elucidate the molecular mechanism underlying the synergistic antitumor activity of ECD NPs-loaded hydrogel, the cascade of events from cellular internalization to apoptotic execution was systematically investigated. Cellular uptake assays revealed that ECD NPs-loaded hydrogel facilitated efficient Cu^2+^ delivery, with intracellular Cu^2+^ levels reaching 6.4 nmol/10^6^ cells in SiHa and 5.8 nmol/10^6^ cells in HeLa cells (Figure 4A). These values were comparable to those in the EC NPs-loaded hydrogel group, indicating that DATS loading did not impair cellular internalization. Following Cu^2+^ internalization, GSH levels dropped significantly to 85.0% in SiHa and 80.0% in HeLa cells relative to untreated controls (Figure 4B). Intracellular ROS levels were substantially elevated following ECD NPs-loaded hydrogel treatment, as evidenced by enhanced green fluorescence relative to the control group (Figure 4C). The coupled GSH consumption and ROS accumulation indicate disruption of intracellular redox homeostasis. Mechanistically, Cu^2+^ released from ECD NPs-loaded hydrogel in the acidic lysosomal compartment catalyzes Fenton-like reactions to generate hydroxyl radicals (·OH) while oxidizing GSH to glutathione disulfide (GSSG) [62]. This dual action depletes the antioxidant reservoir and amplifies oxidative stress. Similar Cu^2+^-based nanoplatforms have been reported to produce ROS alongside GSH depletion, thereby accumulating oxidative stress for catalytic therapy [42,43].

The expression of key regulatory proteins in the mitochondrial pathway was quantitatively assessed. As shown in Figure 4D–F, ECD NPs-loaded hydrogel treatment significantly upregulated the pro-apoptotic protein BAX and downregulated the anti-apoptotic protein Bcl-2 in both cell lines, leading to a markedly elevated BAX/Bcl-2 ratio. BAX expression in SiHa cells reached 1.19-fold in the EC NPs-loaded hydrogel group, 1.30-fold in the DATS-loaded hydrogel group, and 1.41-fold in the ECD NPs-loaded hydrogel group versus the control. Bcl-2 levels dropped to 0.78, 0.57, and 0.49 of the control value, respectively. The corresponding BAX/Bcl-2 ratios rose to 1.52 in the EC NPs-loaded hydrogel group, 2.27 in the DATS-loaded hydrogel group, and 2.87 in the ECD NPs-loaded hydrogel group relative to the control. A similar trend was observed in the HeLa cells. Caspase-3/7 activity followed the same hierarchical order (Figure 4G). Caspase-3/7 activity in the SiHa cells reached 1.05-fold in the EC NPs-loaded hydrogel group, 1.22-fold in the DATS-loaded hydrogel group, and 1.44-fold in the ECD NPs-loaded hydrogel group versus the control, with the ECD NPs-loaded hydrogel group showing the strongest activation. These results confirm that ECD NPs-loaded hydrogel triggers apoptosis through the intrinsic mitochondrial pathway by disrupting the BAX/Bcl-2 balance and subsequently activating Caspase-3/7. Comparable observations have been reported for copper-based nanoparticles, which induce mitochondrial dysfunction and activate caspase-dependent apoptosis via BAX/Bcl-2 modulation [63]. Collectively, these findings establish a coherent mechanistic cascade. Upon cellular internalization, ECD NPs-loaded hydrogel is delivered into the acidic lysosomal compartment, where the MPN structure disassembles and releases Cu^2+^ and DATS simultaneously. The released Cu^2+^ catalyzes Fenton-like reactions to generate ·OH and oxidizes GSH to GSSG, depleting the antioxidant reservoir and inducing oxidative stress. Concurrently, DATS acts on the mitochondrial pathway by upregulating BAX and downregulating Bcl-2, leading to an elevated BAX/Bcl-2 ratio. This imbalance triggers activation of executioner caspase-3/7, ultimately culminating in mitochondrial apoptosis.

### 3.5. In Vivo Antitumor Efficacy and Biocompatibility in an Orthotopic Cervical Cancer Model

**Figure 5:**
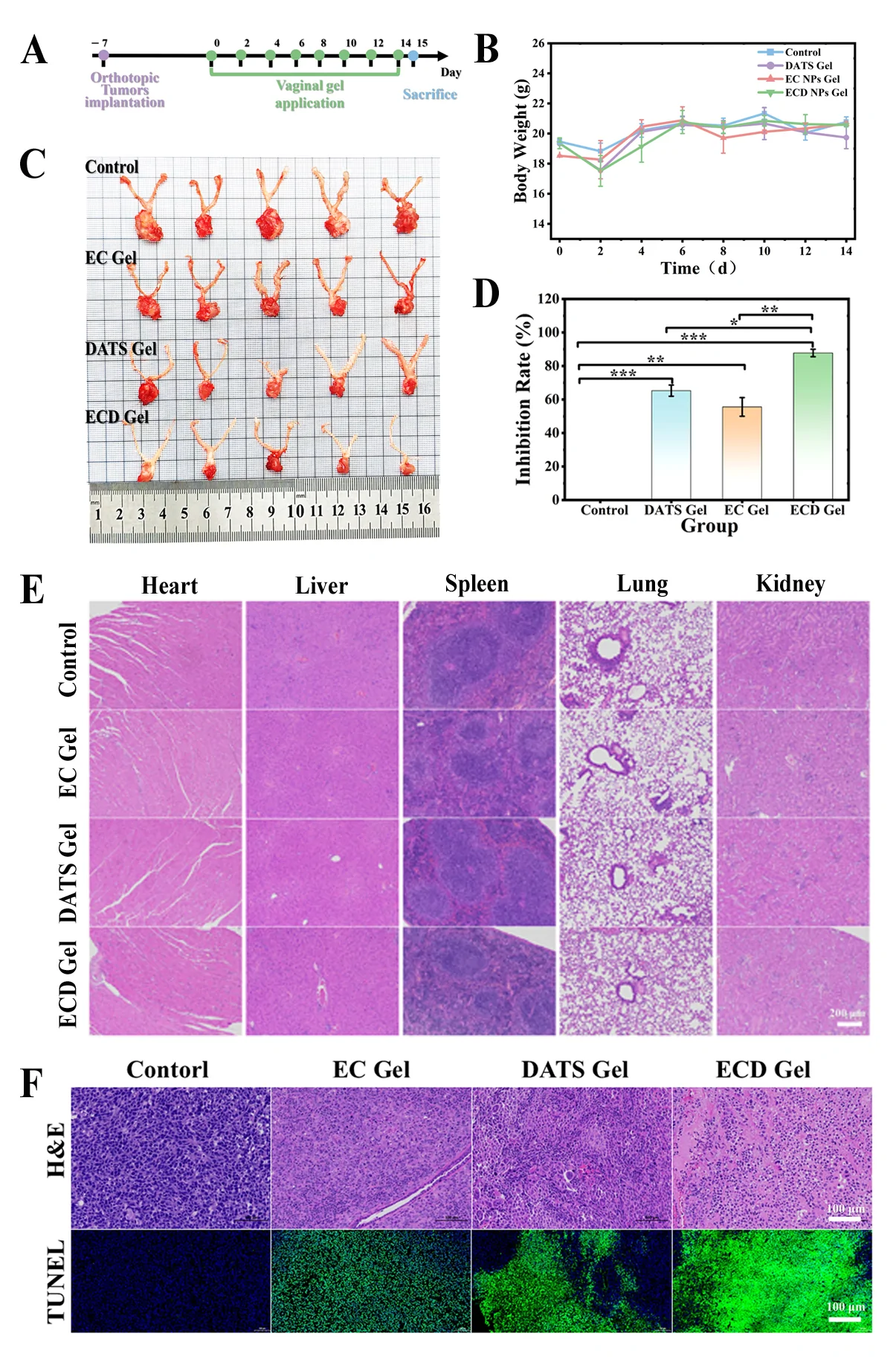
The in vivo antitumor efficacy and biosafety of ECD NPs-loaded hydrogel in an orthotopic cervical cancer mouse model. (**A**) A schematic illustration of the orthotopic cervicovaginal tumor model. (**B**) The body weight changes of the mice during the 15-day treatment period. (**C**) Representative photographs of tumor tissues from each group after treatment. (**D**) Tumor inhibition rates of each group (*n* = 5). (**E**) H&E staining images of major organs (heart, liver, spleen, lung, and kidney) from each group (scale bar = 100 μm). (**F**) H&E staining (upper panel) and TUNEL staining (lower panel) of tumor tissues from each group (scale bar = 100 μm). Data are presented as mean ± SD. * *p* < 0.05, ** *p* < 0.01, *** *p* < 0.001 vs. untreated by Tukey’s HSD test after one-way ANOVA.

The therapeutic potential of the ECD NPs hydrogel was evaluated in an orthotopic cervical cancer model. The schedule of the experiment is shown in Figure 5A. Body weights remained stable in the ECD NPs-loaded hydrogel group throughout the treatment period, while the control group showed progressive weight loss (Figure 5B). No mortality or behavioral abnormalities were observed in any treatment group. The ECD NPs-loaded hydrogel group exhibited a marked reduction in tumor burden compared with the control and monotherapy groups (Figure 5C). Quantitatively, the ECD NPs-loaded hydrogel achieved a tumor inhibition rate of 87.81% (Figure 5D). This was significantly higher than the rates achieved by the free DATS-loaded hydrogel (65.3%) and the EC NPs-loaded hydrogel (55.62%), confirming the potent enhanced antitumor effect of the nanoplatform in vivo.

Histopathological examination was performed on major organs, including the heart, liver, spleen, lung, and kidney. No pathological abnormalities were observed in any treatment group. Inflammation, necrosis, and fibrosis were not detected (Figure 5E). This systemic safety profile aligns with the favorable hemocompatibility observed in vitro. At the tumor tissue level, H&E staining showed extensive nuclear fragmentation and karyolysis in the ECD NPs-loaded hydrogel group, indicative of massive cell death (Figure 5F, upper panel). TUNEL staining showed widespread apoptotic nuclei (brown staining) throughout tumor sections in the ECD NPs group. By comparison, only scattered positive signals were detected in the control group (Figure 5F, lower panel). These results collectively demonstrate that the ECD NPs-loaded thermosensitive hydrogel effectively eradicates orthotopic cervical tumors by inducing apoptosis while maintaining acceptable biocompatibility.

## 4. Discussion

### 4.1. ECD NPs as a Dual-Functional Carrier for DATS Delivery

The selection of ECH as the polyphenol ligand represents a departure from conventional MPN designs. Most reported MPNs rely on polyphenols such as tannic acid, gallic acid, or epigallocatechin gallate, which function primarily as structural scaffolds with limited intrinsic therapeutic activity [44,45,46]. Recent reviews have noted that curcumin, tannic acid, and EGCG have dominated the field, whereas MPNs incorporating polyphenols with intrinsic bioactivity remain scarce [35,64]. ECH, by contrast, is a phenylethanoid glycoside with documented antitumor activity against cervical cancer through Wnt/β-catenin pathway modulation [53]. Coordination between the catechol moieties of ECH and Cu^2+^ was achieved under mild aqueous conditions, eliminating the need for organic solvents or harsh synthesis. This one-step assembly approach compares favorably with multi-component copper-based MPNs systems that often require more complex fabrication procedures.

The high DATS loading capacity (27.6%) is attributable to the porous network structure of the MPN matrix, which provides ample space for hydrophobic drug encapsulation. Copper-based MPNs have been increasingly recognized for their intrinsic anticancer activity beyond serving as delivery vehicles. The present system thus integrates the pharmacological properties of both the metal ion and the polyphenol ligand. This aligns with the emerging paradigm of using MPNs as multifunctional therapeutic platforms rather than passive delivery vehicles.

### 4.2. pH-Responsive Thermosensitive Hydrogel for Vaginal Administration

The ECD NPs hydrogel addresses this limitation through the pH-dependent dissociation of the ECH-copper coordination network. MPNs are inherently responsive to acidic stimuli, as their coordination bonds disassemble in the tumor microenvironment or intracellular lysosomes, enabling targeted payload release [65]. At vaginal pH 4.5, only 32.4% DATS was released, suggesting limited premature leakage in the healthy vaginal environment. At tumor pH 6.5 and lysosomal pH 5.6, release increased markedly to 64.8% and 80.9%, respectively, confirming responsiveness to acidic conditions along the delivery route. This stimulus-responsive behavior minimizes off-target exposure to healthy vaginal epithelium and systemic circulation.

The gelation temperature of 36.7 °C ensures rapid transition from sol to gel upon vaginal administration. Poloxamer-based in situ gels have been widely investigated for vaginal delivery due to their ability to form depots at body temperature. However, most reported systems function as passive retention matrices without active responsiveness to the TME [66,67]. The present system, integrating pH-responsive MPN dissociation with thermosensitive gelation, represents a substantive advancement over such passive formulations. The incorporation of HPMC further enhances mucoadhesive properties, promoting prolonged retention in the dynamic vaginal environment.

### 4.3. Cu^2+^-Mediated Oxidative Stress and DATS-Induced Mitochondrial Apoptosis

The mechanistic findings delineate a complementary cascade wherein Cu^2+^-mediated CDT and DATS-induced mitochondrial apoptosis converge to disrupt redox equilibrium [68]. ECD NPs are internalized into the acidic lysosomal compartment, where the MPN structure disassembles to release Cu^2+^ and DATS. Cu^2+^ then participates in two parallel reactions: conversion of H_2_O_2_ into ·OH via Fenton-like chemistry, and oxidation of GSH to GSSG. This GSH-consuming Fenton-like cycle has been demonstrated in multiple copper-based nanoplatforms. The depletion of GSH lowers the cellular antioxidant threshold, rendering cancer cells more susceptible to oxidative damage.

DATS operates through a mechanistically distinct but therapeutically complementary pathway. As a garlic-derived organosulfur compound, DATS has been shown to induce mitochondria-dependent apoptosis across multiple cancer cell lines [20,21]. The hallmark of this pathway is an increased Bax/Bcl-2 ratio, which triggers mitochondrial membrane depolarization and subsequent activation of caspase-9 and caspase-3. The convergence of these two pathways is central to the system’s advantage. Cu^2+^-mediated CDT intensifies ROS production while simultaneously depleting the antioxidant defenses that would otherwise neutralize DATS-induced oxidative stress. DATS, in turn, amplifies the apoptotic signal through BAX/Bcl-2 dysregulation. This dual-axis intervention targets both ROS generation and ROS disposal. This design addresses a key limitation of single-agent therapies, namely the compensatory upregulation of antioxidant systems that frequently drives therapeutic resistance [69,70].

### 4.4. Clinical Translation Potential for Local Cervical Cancer Therapy

The use of natural products as core components offers a favorable safety profile compared to synthetic chemotherapeutics or inorganic nanomaterials. ECH and DATS are derived from dietary or medicinal plants with documented long-term human exposure, which reduces the risk of unexpected toxicity. The vaginal route of administration further minimizes systemic exposure, as reflected by the absence of pathological damage to major organs and stable body weights throughout the treatment period [71].

This localized approach may be of particular interest for younger patients, in whom fertility preservation is often a key consideration. Current radical treatments, including trachelectomy and platinum-based chemotherapy, are associated with surgical morbidity, ovarian toxicity, and infertility. Vaginal delivery offers a theoretical advantage by reducing systemic drug exposure, which may help minimize off-target effects [7]. The hydrogel is designed for practical use, with a sol–gel transition at 36.7 °C that allows administration as a liquid at room temperature and rapid gelation upon contact with vaginal tissue. This feature, together with the pH-responsive release profile, supports sustained local drug levels with minimal leakage.

### 4.5. Limitations and Future Directions

Several limitations warrant consideration. The U14-based orthotopic model does not fully replicate human cervical cancer, necessitating validation in human-derived xenograft or HPV-transgenic models. The independent contribution of ECH remains unclear and requires pathway-specific mechanistic dissection. Tumor selectivity was not assessed using normal cervical or vaginal epithelial cells, and this aspect also remains to be addressed. Additionally, copper-mediated hydroxyl radical generation and lysosomal drug release were not directly verified, and these mechanistic aspects remain to be established. Formulation optimization, including lyophilized precursor development and long-term stability studies, is needed to facilitate clinical translation. Immunomodulatory effects and reproductive toxicity following extended dosing also merit systematic investigation. These ongoing efforts will further inform the clinical potential of this platform.

## 5. Conclusions

This study developed an ECD NPs-loaded thermosensitive hydrogel for localized cervical cancer therapy, with innovations spanning formulation design, mechanistic elucidation, and translational validation. At the formulation level, ECH was employed as a dual-functional polyphenol ligand for Cu^2+^ coordination, departing from conventional inert MPN scaffolds. The resulting pH-responsive system achieved high DATS loading and triggered release, addressing the limitations of passive retention hydrogels. Mechanistically, the platform established a synergistic cascade: Cu^2+^-mediated GSH depletion and ROS generation cooperated with DATS-induced BAX/Bcl-2 dysregulation and caspase-3/7 activation, effectively overcoming redox resistance in cervical cancer cells. In an orthotopic mouse model, the hydrogel achieved an 87.81% tumor inhibition rate with no evident systemic toxicity, supporting its potential as a localized therapy with reduced systemic toxicity. Beyond this specific application, the work demonstrates that functional polyphenol selection can transform MPNs from passive carriers into active therapeutic participants, offering a generalizable strategy for synergistic cancer treatment.

## Data Availability

Data will be made available on request.

## Supplementary Materials

The following supporting information can be downloaded at https://www.mdpi.com/article/10.3390/pharmaceutics18091126/s1, Figure S1: Representative HPLC chromatograms. (A) DATS standard. (B) EC NPs. (C) ECD NPs; Figure S2: Optimization of EC NPs preparation conditions. (A) Effects of different preparation methods (One-step Stirring, Ultrasonication (Bath), and Cell Homogenizer (Probe)) on the particle size and PDI of EC NPs. (B) Effects of different ECH-to-Cu^2+^ molar ratios (1:1 to 6:1) on the particle size and PDI of EC NPs. Data are presented as mean ± SD (n = 3); Table S1: Validation parameters of the HPLC method for DATS determination; Table S2: Gelation temperature of the thermosensitive hydrogel at different drug loading concentrations.

## Abbreviations

The following abbreviations are used in this manuscript:

DATS	Diallyl trisulfide
MPNs	Metal–phenolic networks
ECH	Echinacoside
EC NPs	ECH-copper nanoparticles
ECD NPs	ECH-copper-DATS nanoparticles
