# Supplementary material for: Study on Echinacoside-Copper Metal–Phenolic Networks Hydrogel System Loaded with Diallyl Trisulfide for Local Treatment of Cervical Cancer
Source: Pharmaceutics. 2026 Sep 8;18(9):1126. doi: 10.3390/pharmaceutics18091126 (PMC13610057; doi:10.3390/pharmaceutics18091126)
Supplement: Supplementary file 1 [file pharmaceutics-18-01126-s001.zip › pharmaceutics-4497463-supplementary.pdf]

Study on Echinacoside-Copper Metal-Phenolic Networks Hydrogel System Loaded with Diallyl Trisulfide for Local Treatment of Cervical Cancer

Na Zhao[1,†], Xiaoqian Zhang [1,†], Jing Luo[1], Xiaoyue Zhang[1], Yonghong Zhao[1], Jiang Liu[1], Le Li[1], Chenglin Hong[1,*], Shiguo Sun [1,2] *

[1] School of Pharmacy/Key Laboratory of Xinjiang Phytomedicine Resource and Utilization Ministry of Education/Institute for Safflower Industry Research, Shihezi University, Shihezi, Xinjiang, 832002, China.; zhaona@shzu.edu.cn (N.Z.); 20232015021@stu.shzu.edu.cn (X.Z.); 15699399805@163.com (J.L.); 15160973823@163.com (X.Z.); liujiang@shzu.edu.cn (J.L.); teacher.lee@foxmail.com (L.L.);

[2] College of Chemistry & Pharmacy, Northwest A&F University, Yangling 712100, China

* **Correspondence**: hcl_tea@shzu.edu.cn (C. H.); sunsg@nwsuaf.edu.cn (S. S.); +86-0993-2057-006 (C. H.);Tel.: +86-029-8709-2303 (S.S.)

† These authors contributed equally to this work.

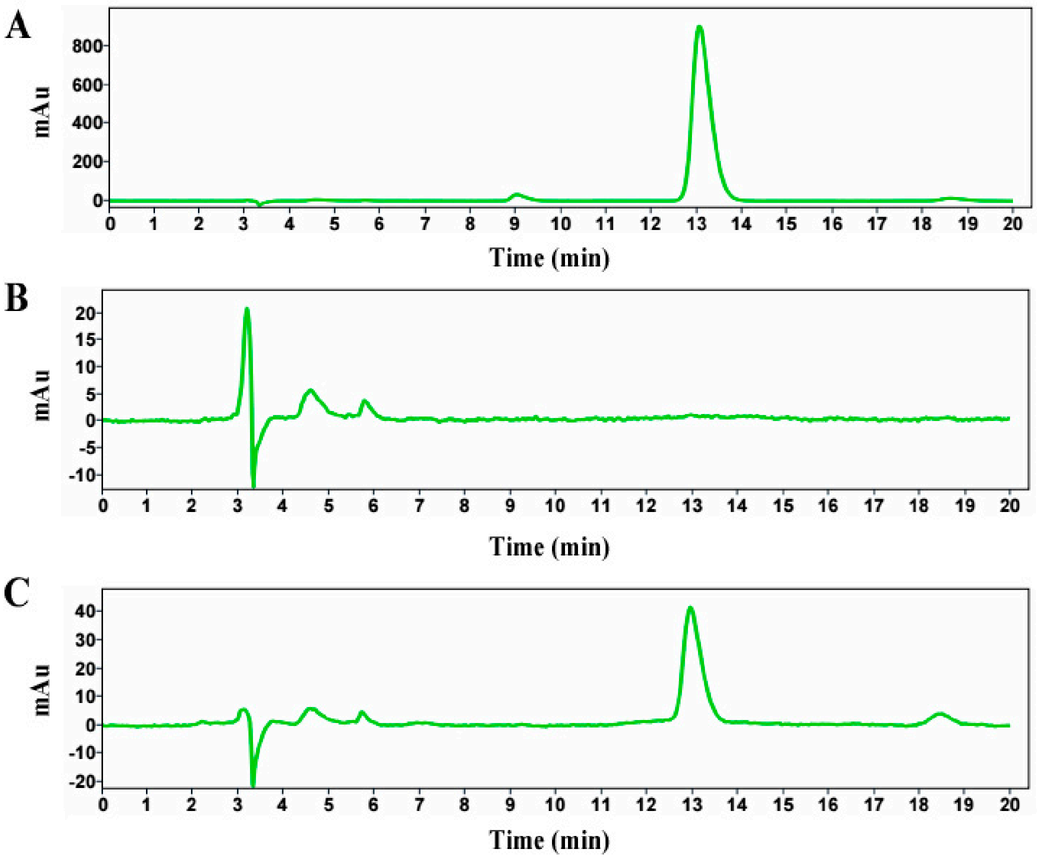

Supplementary Figure S1. Representative HPLC chromatograms. (A) DATS standard solution; (B) EC NPs (without drug loading); (C) ECD NPs (DATS-loaded nanoparticles). The chromatographic conditions were as follows: an octadecylsilyl silica gel column (250 mm × 4.6 mm, 5 μm) as the stationary phase, a mobile phase consisting of methanol-0.1% formic acid solution (75:25, v/v), a detection wavelength of 210 nm, a flow rate of 1.0 mL/min, and a column temperature of 30 °C.

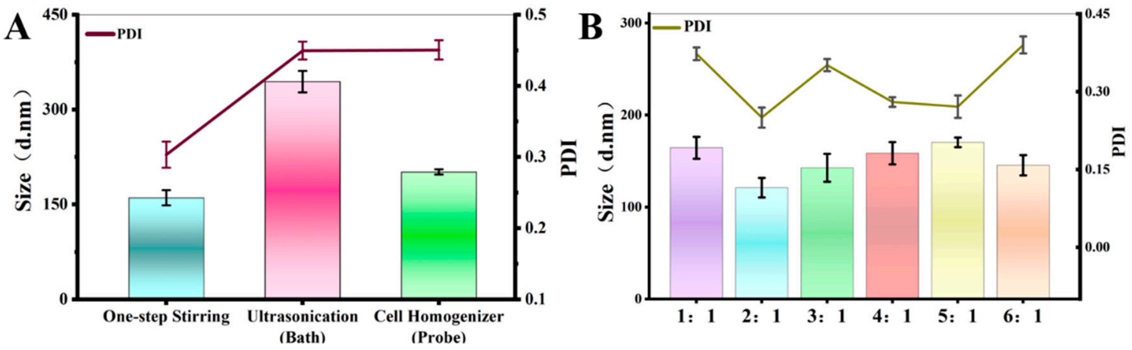

Supplementary Figure S2. Optimization of EC NPs preparation conditions. (A) Effects of different preparation methods (One-step Stirring, Ultrasonication (Bath), and Cell Homogenizer (Probe)) on the particle size and PDI of EC NPs. (B) Effects of different ECH-to-Cu^{2+} molar ratios (1:1 to 6:1) on the particle size and PDI of EC NPs. Bars represent particle size (nm), and square dots represent the Polydispersity Index (PDI). Data are presented as mean ± SD (n = 3).

Supplementary Table S1. Validation parameters of the HPLC method for DATS determination.

Parameter	Result	Acceptance criteria
Linearity range	10.4-166.4 μg/mL	-
Correlation coefficient (R^2)	0.9998	≥ 0.995
Precision (RSD, n=6)	0.08%	≤ 2.0%
Repeatability (RSD, n=6)	0.99%	≤ 2.0%
Stability (RSD over 24 h)	0.55%	≤ 2.0%
Recovery (n=3)	97.3-103.2%	95.0-105.0%
Recovery RSD	1.81%	≤ 2.0%

Supplementary Table S2 Gelation temperature of the thermosensitive hydrogel at different drug loading concentrations.

Drug loading concentration (mg/mL)	Gelation temperature (°C)
0	37.0±0.20
2	35.5±0.10
2.5	35.0±0.17
3	36.0±0.20
3.5	36.7±0.10
4	36.2±0.17

Note: All experiments were performed in triplicate (n=3). Data are expressed as mean ± standard deviation (SD).
